# Supplementary material for: Glycyl-tRNA sequestration is a unifying mechanism underlying GARS1-associated peripheral neuropathy
Source: Nucleic Acids Res. 2025 Mar 22;53(6):gkaf201. doi: 10.1093/nar/gkaf201 (PMC11928938; doi:10.1093/nar/gkaf201)
Supplement: gkaf201_Supplemental_File [file gkaf201_supplemental_file.pdf]

Supplementary Information for

Glycyl-tRNA sequestration is a unifying mechanism underlying *GARS1*-associated peripheral neuropathy

Natalia Mora[1#*], Erik F.J. Slot[1#], Vanessa Lewandowski[2], Maria P. Menafra[1], Moushami Mallik[1], Pasal van Lith[1], Céline Sijlmans[1], Nick van Bakel[1], Zoya Ignatova[2], Erik Storkebaum[1*]

AFFILIATIONS

[1]Molecular Neurobiology Laboratory, Donders Institute for Brain, Cognition and Behaviour and Faculty of Science, Radboud University, Nijmegen, Netherlands

[2]Department of Biochemistry and Molecular Biology, Hamburg University, Hamburg, Germany

#The first two authors contributed equally to this work

*To whom correspondence should be addressed:

Natalia Mora: natalia.moragarcia@donders.ru.nl

Erik Storkebaum: erik.storkebaum@donders.ru.nl
Phone: +31 6 25 76 60 73

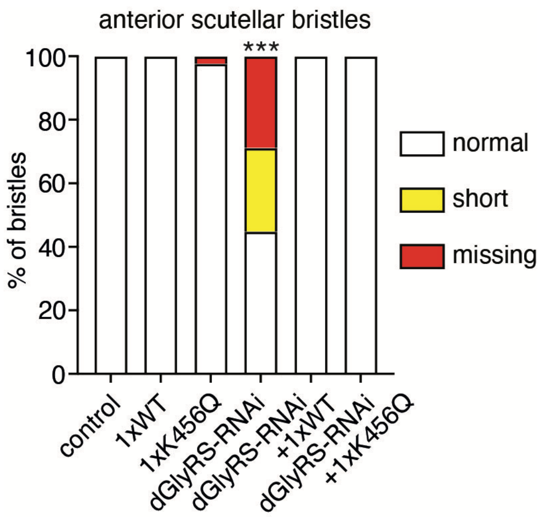

Supplementary Figure 1: Quantification of the percentage of normal (long), short, or missing anterior scutellar bristles in the presence or absence of dGlyRS knock-down, and with or without simultaneous co-expression of hGlyRS-WT or hGlyRS-K456Q. n = 36 to 118 bristles per genotype; ***p<0.0001 by Fisher's exact test.

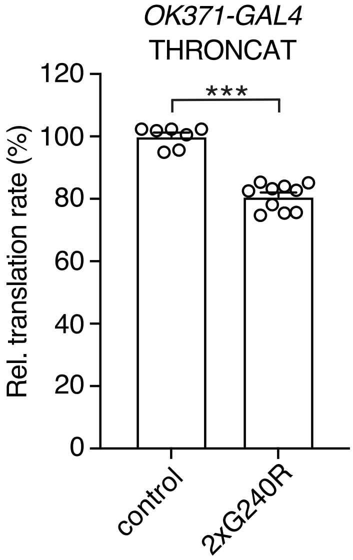

Supplementary Figure 2: Relative translation rate (% of driver-only control) as determined by THRONCAT in motor neurons (*OK371-GAL4*) of larvae expressing hGlyRS-G240R. n = 7 to 12 animals per genotype; ***p<0.0001 by Mann-Whitney test. The control data points are the same as in Figure 5E, because the 2xG240R condition was included in the same larger experiment.

oligo	sequence
GARS-EagI_FW	GCATAACGGCCGatgccctctccgcgtccag
GARS-XbaI_REV	CCATGACTCTAGAttattcctcgattgtctctttttaccagtctcttgc
S211F_muta_FW	AATGTAAAATtTCCCATTACTGG
S211F_muta_REV	ATAGTTCACAAAAAGATCCG
H418R_muta_FW	CTCTCCTGTCgTGCACGAGCC
H418R_muta_REV	GTCATAACAGGAACGATCAGCAC
K456Q_muta_FW	GGCATATAAGcAGGATGCAAAAC
K456Q_muta_REV	TTACCAATTGCTCCCTTAC

Supplementary Table 1: Sequences of primers used for cloning and mutagenesis of human GARS1 cDNA.

target	oligo	sequence
dGlyRS mRNA	forward	CACTCTAAAGGAACCGCATACGG
	reverse	ACACTCTCCCAGGTGGTTCTCG
hGlyRS mRNA	forward	GCACCTCTGAGGCTAGCAGT
	reverse	GAGCTCAGCCACTGCTTTGT
Beta-actin mRNA	forward	GCCATGTATGTGGCCATCC
	reverse	CGACCAGCCAGATCCAGAC
Alpha-tubulin mRNA	forward	CCATCCTGACCACCCACACC
	reverse	GACGGTTCAGGTTGGTG

Supplementary Table 2: Sequences of primers used for quantitative real-time PCR (qPCR).

GlyRS mutation	effect on net GlyRS charge	evolutionary conservation of mutated AA residue	segregation with disease in a family	aminoacylation activity	protein synthesis	developmental lethality induced by ubiquitous expression	impaired climbing behavior	reduced dendritic coverage of multidendritic sensory neurons	references
WT	=	NA	NA	=	=	-	-	-	
E71G	+1	yeast	yes	=	↓↓	++	++	+	(1-4)
S211F	=	C. elegans	yes	↓↓↓	↓↓↓	+++	++	++	(5,6), this study
G240R	+1	Drosophila	yes	↓↓	↓↓↓	+++	+++	++	(1-4,6)
H418R	=	yeast	yes	↓↓	↓↓↓	+++	++	++	(2,6,7), this study
K456Q	-1	yeast	no	=	=	-	-	-	(8), this study
G526R	+1	yeast	yes	↓↓↓	↓↓↓	+++	+++	++	(1-4,6,9)

Supplementary Table 3: Characteristics of PN-GlyRS variants used in this study. The effect of PN-GlyRS variants on net GlyRS protein charge, aminoacylation activity, evolutionary conservation of the affected amino acid residues, and genetic evidence to support disease causality are shown. Segregation of the mutation with disease in a family provides stronger genetic evidence than mutations found in single patients. Furthermore, a summary of peripheral neuropathy-like phenotypes in *Drosophila* PN-GlyRS models is provided, including the effect of expression of PN-GlyRS variants on protein synthesis in motor neurons, developmental lethality induced by ubiquitous expression, impaired adult climbing behavior induced by motor neuron (*OK371-GAL4*) expression as

evaluated in the negative geotaxis assay, reduced dendritic coverage of class IV multidendritic sensory neurons upon selective expression in these neurons (*ppk-GAL4*). Symbols: = not altered; ↓ mild reduction; ↓↓ moderate reduction; ↓↓↓ severe reduction; - no phenotype; + mild phenotype; ++ moderate phenotype; +++ severe phenotype. AA: amino acid; NA: not applicable; ND: not determined.

References

1. Antonellis, A., Ellsworth, R.E., Sambuughin, N., Puls, I., Abel, A., Lee-Lin, S.Q., Jordanova, A., Kremensky, I., Christodoulou, K., Middleton, L.T. *et al.* (2003) Glycyl tRNA synthetase mutations in Charcot-Marie-Tooth disease type 2D and distal spinal muscular atrophy type V. *Am J Hum Genet*, **72**, 1293-1299.
2. Antonellis, A., Lee-Lin, S.Q., Wasterlain, A., Leo, P., Quezado, M., Goldfarb, L.G., Myung, K., Burgess, S., Fischbeck, K.H. and Green, E.D. (2006) Functional analyses of glycyl-tRNA synthetase mutations suggest a key role for tRNA-charging enzymes in peripheral axons. *J Neurosci*, **26**, 10397-10406.
3. Nangle, L.A., Zhang, W., Xie, W., Yang, X.L. and Schimmel, P. (2007) Charcot-Marie-Tooth disease-associated mutant tRNA synthetases linked to altered dimer interface and neurite distribution defect. *Proc Natl Acad Sci U S A*, **104**, 11239-11244.
4. Niehues, S., Bussmann, J., Steffes, G., Erdmann, I., Kohrer, C., Sun, L., Wagner, M., Schafer, K., Wang, G., Koerdt, S.N. *et al.* (2015) Impaired protein translation in Drosophila models for Charcot-Marie-Tooth neuropathy caused by mutant tRNA synthetases. *Nature communications*, **6**, 7520.
5. Lee, H.J., Park, J., Nakhro, K., Park, J.M., Hur, Y.M., Choi, B.O. and Chung, K.W. (2012) Two novel mutations of GARS in Korean families with distal hereditary motor neuropathy type V. *J Peripher Nerv Syst*, **17**, 418-421.
6. Griffin, L.B., Sakaguchi, R., McGuigan, D., Gonzalez, M.A., Searby, C., Zuchner, S., Hou, Y.M. and Antonellis, A. (2014) Impaired function is a common feature of neuropathy-associated glycyl-tRNA synthetase mutations. *Hum Mutat*, **35**, 1363-1371.
7. Sivakumar, K., Kyriakides, T., Puls, I., Nicholson, G.A., Funalot, B., Antonellis, A., Sambuughin, N., Christodoulou, K., Beggs, J.L., Zamba-Papanicolaou, E. *et al.* (2005) Phenotypic spectrum of disorders associated with glycyl-tRNA synthetase mutations. *Brain*, **128**, 2304-2314.
8. Forrester, N., Rattihalli, R., Horvath, R., Maggi, L., Manzur, A., Fuller, G., Gutowski, N., Rankin, J., Dick, D., Buxton, C. *et al.* (2020) Clinical and Genetic Features in a Series of Eight Unrelated Patients with Neuropathy Due to Glycyl-tRNA Synthetase (GARS) Variants. *J Neuromuscul Dis*, **7**, 137-143.
9. Dubourg, O., Azzedine, H., Yaou, R.B., Pouget, J., Barois, A., Meininger, V., Bouteiller, D., Ruberg, M., Brice, A. and LeGuern, E. (2006) The G526R glycyl-tRNA synthetase gene mutation in distal hereditary motor neuropathy type V. *Neurology*, **66**, 1721-1726.
